# Physiology labs during a pandemic: What did we learn?

**DOI:** 10.1152/advan.00022.2021

**Published:** 2021-12-01

**Authors:** Xinnian Chen, Catherine B. Kirn-Safran, Talitha van der Meulen, Karen L. Myhr, Alan H. Savitzky, Melissa A. Fleegal-DeMotta

**Affiliations:** ^1^Department of Physiology and Neurobiology, University of Connecticut, Storrs, Connecticut; ^2^Department of Biology, Widener University, Chester, Pennsylvania; ^3^Department of Neurobiology, Physiology, and Behavior, College of Biological Sciences, University of California, Davis, Davis, California; ^4^Department of Biological Sciences, Wayne State University, Detroit, Michigan; ^5^Department of Biology, Utah State University, Logan, Utah; ^6^Department of Science and Mathematics, Biology, Clarke University, Dubuque, Iowa

**Keywords:** COVID-19, online learning, physiology laboratory, remote instruction, scientific teaching

## Abstract

This article captures a collective reflection on the successes and challenges we experienced when teaching physiology laboratories online during the COVID-19 pandemic. Physiology instructors from six institutions discussed their own efforts to redesign meaningful physiology laboratories that could be taught remotely, as the nation scrambled to respond to the sudden shift out of the classroom. Despite the complexity of this task, clear themes emerged as our courses transitioned to an online format in spring 2020 and were solidified in the fall of 2020. This article reflects on the history, features, benefits, and challenges of current laboratory teaching when applying a scientific teaching approach to facilitate the redesign process. We believe online networks like ours can facilitate information sharing, promote innovations, and provide support for instructors. The insights we gained through this collaboration will influence our thinking about the future of the physiology lab, whether online or in person.

## INTRODUCTION

The addition of a laboratory experience to parallel or augment a course’s lecture content is often viewed as the gold standard for pedagogy in the natural sciences (1). This holds true for introductory survey courses for first-year students and for upper-division courses that serve a small subset of majors, where students learn to practice scientific skills and solidify their career interests. The sudden appearance and stubborn persistence of the novel coronavirus disease 2019 (COVID-19), however, has had a dramatic impact on the teaching of laboratory courses and has forced a reckoning over what skills are essential for scientists, what students find interesting and important for their careers, and how these skills can best be taught in a largely remote learning environment. Here, we explore the history of laboratory teaching and some of the philosophical underpinnings and practical consequences of backward design that inform faculty plans for remote laboratory instruction in the biological sciences, with an emphasis on the teaching of comparative and human physiology. In this article, we take a broad view of what laboratory courses could be. We ask to what degree the responses demanded by COVID-19 might foretell more lasting—and beneficial—changes in laboratory pedagogy.

### A Brief History of Laboratory Teaching

The Western tradition of science began as the pastime of wealthy elites, and as science moved into the academic realm formal training led to a class of professional, but nonetheless elite, practitioners. In the late nineteenth and twentieth centuries, agricultural demands and the First and Second World Wars drove biological research (2). During World War II rapid technological advances occurred in all fields of science, including improvements in light and electron microscopy, spectroscopy, experimental and medical microbiology, and environmental monitoring. In the postwar years, biology courses were increasingly driven by the available technology, often involving “experiments” that were essentially cookbook applications of an instrument, guaranteed to deliver an expected result. Although the instruments themselves evolved, little about the fundamental nature of such pro forma laboratory exercises changed. Institutions and governments invested heavily in the technology that supported these carefully calibrated laboratory activities, and students emerged from them trained to apply the instrumentation that had become ubiquitous in biomedical research and clinical settings. What was often missing, however, was careful consideration of the fundamental questions that underpinned such laboratory activities. The laboratory experience simply demonstrated well-known principles with the latest instrumentation. The laboratory manual laid out a standard question, and the students put the instruments through their paces, arriving at a predetermined answer (3).

In the early years of the twenty-first century, educators began to voice concerns that the laboratory experience had become decoupled from the “process of science,” the steps by which a hypothesis is generated, tested, refuted or refined, and tested again (4). These concerns led to workshops that culminated in the landmark publication of *Vision and Change in Undergraduate Biology Education: A Call to Action* (5). That report lent authority to growing efforts to take undergraduate biology courses beyond cookbook laboratory activities to more “authentic” research experiences such as course-based undergraduate research experiences (CUREs). Organizations such as the Partnership for Undergraduate Life Sciences Education (PULSE) and the National Institute on Scientific Teaching (NIST), formerly known as the Summer Institute on Scientific Teaching (SI), emerged in the wake of these reports. Additional working groups and reports soon followed, and the broader movement that emerged brought welcome change to many biology classrooms, focusing laboratory courses on training students to ask testable questions and to devise studies to address them (6). In those efforts, laboratory classes were no longer driven simply by the instrumentation present in the classroom. Instead, the instrumentation was employed in service to the question, just as it is in a research laboratory. Specific skills were mastered while addressing student-generated research questions, and students learned why the technology of science was needed (and why it can be a limiting factor in answering such questions).

### Forced Shift in Laboratory Teaching in Response to the Pandemic

Just as more biology laboratory instructors realized the need to change and were progressively adopting more effective pedagogies, the COVID-19 pandemic exploded on the scene. Although the pandemic created many challenges, it also moved the laboratory course one step closer to a complete transformation. Remote laboratories required that research questions be asked and answered largely in the absence of state-of-the-art instrumentation. Although several companies responded by making home kits that students use to collect physiological data outside of the laboratory, we found that there were many logistical issues, such as supervision, liability, cost, and shipping, associated with such kits. These issues are especially challenging for large-enrollment courses and for departments and institutions facing financial shortfalls due to COVID-19. Here, we reflect on our collective experiences with physiology laboratories untethered from the instrumentation that we had previously relied upon.

### Applying Backward Design to the Development of Online Labs

When the COVID-19 pandemic hit in spring 2020, institutions and their faculty were forced to pivot suddenly to an online format, disrupting the norm of our practices and constraining us to make spur-of-the-moment decisions for the coming weeks and beyond. Sharing goals and experiences as a group that were already trained in scientific teaching helped us support each other and clarify what worked online as the pandemic and online laboratories continued. After the abrupt pivot, the members of our group took a step back over the summer of 2020, recognizing the importance of redesigning our physiology laboratories based on learning objectives we wanted students to achieve, rather than simply finding alternative laboratory activities we could offer online. To do this, we asked ourselves “What is a lab?” and “What skills and benefits derive from the laboratory experience?” This reflection forced us to evaluate the traditional laboratories we previously expected students to complete and to determine which skills are critical (and which ones are not) for students’ success in future courses and careers. For those skills that are critical but not transferable to an online setting, we asked whether they needed to be taught elsewhere once we return to in-person classes. For example, if one is teaching pre-health professional students and the goal of a physiology laboratory had been to learn to use a stethoscope and measure blood pressure, then those skills would need to be taught in a future course that focuses on clinical elements of patient care. That may require coordination with other faculty members. However, if the goal had been to gather data to explore other concepts, such as how heart sounds change with breathing, then proper use of the stethoscope can be conveyed by demonstration, rather than hands-on practice. Raw data collected by others using a stethoscope can be provided to the students, who can then apply higher-order integration, analysis, and hypothesis testing. Based on our reflection and historical view of laboratory courses (7, 8) and conversations as we continued online, we established seven goals for a physiology laboratory experience, whether the laboratory was taught in an in-person or a synchronous or asynchronous online setting:

1. Reinforce biological and physiological concepts.
2. Utilize theoretical physiological concepts to solve practical questions.
3. Practice the scientific method: hypothesis generation, experimental design, data analysis, refinement, and replication.
4. Improve analytical skills (e.g., data analysis and presentation, figure generation, image analysis, statistical analysis).
5. Develop technical skills in physiology (e.g., consistent and organized data collection, observation of animal behavior, microscopy, and other equipment use).
6. Practice communication skills, both verbal and written.
7. Acquire a basic level of professionalism and apply collaborative skills.

As we moved our laboratory experiences to a strictly online format (synchronous and asynchronous) in the fall of 2020, our goal was to make changes that would align with our list of essential goals. To do this we reviewed and adopted many frameworks related to teaching the components of the scientific process (9–13). One model that was particularly beneficial to guide our discussions was the Buck et al. model (10), which characterizes laboratories into five levels: Level 0, confirmation; Level 0.5, structured inquiry; Level 1, guided inquiry; Level 2, open inquiry; and Level 3, authentic inquiry. In this model, the levels are defined, from an instructor’s point of view, by what instructors do or do not provide to the students for the individual aspects of the laboratory. Another model from Weaver et al. (12) adds the student perspective by including an axis of student responsibility. We combined these two models into one and used it to frame our approach (Table 1). Without the constraints of supervising the use of specific equipment, we were able to provide more support when asking students to take more responsibility for additional elements of the scientific process. We provided guidelines on how to develop procedures, rather than specific procedures and equipment. In this way, we redesigned our laboratories toward open or authentic inquiry from structured or guided inquiry (10). Working backward from goals to assessments and then to assignments/activities in this redesigned, student-centered approach has allowed us to focus on components that directly address our seven goals. Although the strategies adopted by our courses are different, we discovered three common themes.

**Table 1.** Summary of transition to authentic inquiry

	Confirmation	Structured Inquiry	Guided Inquiry	Open Inquiry	Authentic inquiry
Conclusions and communication	Provided	1. Final report on how evidence supports conclusions, and troubleshooting; written, oral, and/or poster 2. Examples, guidelines 3. Feedback from prior scaffolded assignments and check-ins; final feedback; class showcases for authentic audience			
Results analysis	Provided	Provided	1. Lab notebook checks, results and analysis submitted before final report. 2. Lab notebook templates, discussion of analysis and display 3. Notebook and assignment feedback, check-in meetings		
Procedure design	Provided	Provided	Provided	1. Submit design for approval, ongoing reflection and troubleshooting assignments. 2. Project planning, experimental design guidance 3. Discussions of plans before submitting, resubmit if necessary	
Theory/background and problem/question	Provided	Provided	Provided	Provided	1. Question and background submitted for approval 2. Topic ideas, strategies, library resources 3. Rubrics, feedback, meetings to guide questions

The model of Buck et al. (10) notes what is provided or not provided by the instructor for each of the five levels of inquiry (1st row). Where “not provided” was indicated and is now removed from the original table, we included what instructors needed to provide to run each type of lab. The categories are 1. assignments, 2. resources, and 3. student interactions with the instructional staff.

## THEME 1. THE NEED TO ESTABLISH AN INCLUSIVE SCIENTIFIC COMMUNITY

A typical laboratory setting accommodates a relatively limited number of students and usually presents a natural opportunity for students to interact with each other, feel connected, and develop a sense of belonging to their peer community and to the course (14). In an online format, such community building is more challenging; students were hungry for interactions but sometimes felt uncomfortable and reluctant to participate in an online setting (15). Therefore, we spent time creating an online space where students felt it was acceptable, and even encouraged, to make mistakes, voice different opinions, and share their challenges. Those of us teaching synchronously identified icebreaker activities as one of the first steps to engage students in an online discussion. Once students had warmed up to their peers in an online setting, in-depth discussions were more likely to occur.

We also placed a special emphasis on fostering an inclusive scientific community in the online laboratory setting with the use of breakout rooms. We found that students were more likely to participate in breakout room activities when they were with familiar peers, when they felt more welcomed by the group, when more highly structured activities were offered, and when they identified those activities as meaningful. Thus, to help students feel more welcomed, we focused on *1*) creating more communication channels; *2*) facilitating team building by developing communal projects and promoting appropriate and respectful behavior; and *3*) fostering the building of trust among peers, peer-learning assistants (PLAs), teaching assistants (TAs), and professors. Online video-conferencing functions such as chat, annotation tools, or polls were used to engage students during online instruction. To create structure for breakout room activities, we incorporated online collaboration tools that facilitated feedback with other students and instructors. These tools each presented its specific advantages; for example, *1*) when instructors wished to receive real-time independent inputs from students, we successfully used online survey programs; *2*) when giving brief breakout room instructions, online video-conference chat was very effective; and *3*) when longer instructions or worksheets were needed, they were provided in a shareable document (Google Docs, Microsoft Teams and OneDrive, Collaborations in Canvas, etc.). Such tools kept all group members clear on the instructor’s expectations. Likewise, we often included a more defined end product (e.g., reporting out) to increase the structure of these activities. In addition, to better engage our students in these online activities, we needed to be mindful of what interested our students. For example, we noticed when introducing a project related to COVID-19 implications that students often showed enthusiasm in these discussions.

Below is a list of inclusive community-building activities that we used in our courses.

- Learn and use student names from the start
- Icebreakers at the start of the course
- Check-ins at the start of each meeting to make the online experience more personal. Check-in suggestions:
  ∘  Once a week, open with a poll that inquires after their well-being on a scale of 1–5.
  ∘  At the beginning of the session have everyone type a word or phrase in the chat on how they are feeling at that moment or what they are grateful for.
  ∘  Ask questions that anyone can answer about themselves (What is a favorite food in your household at this time of year? What is something good that has come out of the COVID-19 time for you personally? How many midterms do you have this week?).
  ∘  Have a slide with student pets up while waiting for everyone to arrive (ask students to submit photos through the learning management system).
- Small group activities (using breakout rooms) to increase student interaction with each other and the teaching team, achieving a transitory decrease in the student-professor/TA ratio (example: instead of 12:1 reduced to 4:1).
- Use instruction time to pause at regular intervals and call on students by name to explain important concepts in their own words. Index cards with student names to track who has been called on have been instrumental to implement this approach in an online virtual platform (with limited screen space).

Group dynamics and assurance of fair contributions have always been a tricky topic in team-based learning (16). As we realized that it was important, but challenging, to continue team-based learning in a remote learning setting, special attention was paid to promoting constructive behavior and teaching students how to collaborate. To avoid competitive interactions, we deemphasized individual incentives and based assessments largely on the group product. This also created a sense of interdependence among team members. To help students navigate the interdependence of a team successfully for long-term projects, we asked team members to

- Share documents for team projects (in both synchronous and asynchronous classes)
- Write team contracts, to set expectations and increase accountability
- Develop a collaboration rubric for evaluation of self and other team members
- Perform midsemester reflections on team dynamics to increase effectiveness of group activities.

The intent of these activities was to allow students to stay engaged in the online learning modality and to find the balance between intellectual independence and mutually respectful collaboration. The success of the community relied on effective instructor/teaching team training. Typical TA training was modified and focused on discussing and demonstrating inclusive practices, such as failure bow (a practice to acknowledge the learning path) (17, 18) and Tanner’s 21 inclusive strategies (19). We also included discussions on implicit biases, growth mindset, microaggression/microinvalidation, stereotype threat, and their implications in fostering an inclusive classroom (20–22).

## THEME 2. PRACTICE OF SCIENTIFIC PROCESS AND DEVELOPMENT OF ANALYTICAL AND COMMUNICATION SKILLS

As stated above, the traditional in-person laboratory typically focused on students using specific instrumentation to demonstrate physiological principles. These laboratories generally used synchronous laboratory meeting times for learning how to use equipment and for collection of data. Although the pandemic created challenges by reducing the opportunities to learn skills that require expensive, fragile, or large equipment, it also created an opportunity to focus students on learning the scientific process and developing analytical and communication skills.

With the transition to online laboratories, we were able to expand emphasis on skills such as identifying a research question, developing a hypothesis, designing experiments, analyzing data, developing a conclusion, communicating results, and performing peer evaluations (13).

As we switched to more open inquiry laboratories, we needed to provide explicit project directions and resources that could be implemented away from the traditional laboratory setting. One design option was to find alternatives so students could record data at home. For example, in a comparative physiology laboratory, activities could be performed with plants or by video-recording backyard animals or pets exhibiting their normal behaviors. This also meant that we had to rethink how students could collect and analyze data without specific laboratory equipment. Videos of pets could be analyzed with DeepLabCut (http://www.mousemotorlab.org/deeplabcut), and backyard bird or frog calls could be recorded with the BirdNET phone app and analyzed with Raven Lite software (both available from the Cornell Lab of Ornithology). For those courses where specific equipment or resources are required, an option would be to provide students with background information and prerecorded data and ask them to propose a new research question and then develop a hypothesis and design an experiment to answer that question. Each of these types of activities allows students to find an area of interest to explore. For both designs, planning included a list of needed resources, a risk management assessment and mitigation plan, and a timeline. The timeline included a critical path and tasks assigned to team members. As students executed their projects, they each kept a laboratory notebook and submitted reflections on progress and any needed revisions. As students conducted their experiments, they had opportunities to learn how to troubleshoot or change directions within their overarching question when they ran into obstacles. Students learned the value of reading the literature carefully when they were setting up their own protocols. These skills will benefit students as they become scientists or develop projects in any field.

In addition to the early stages of the scientific process, we focused on the analysis and communication stages of the research. Rather than emphasize collection of data during the laboratory period, we structured more time during synchronous online meetings for developing analytical and communication skills.

When focusing on data analysis, we emphasized methods both for analyzing raw data and for statistical analysis, using prepared data sets. Students were given sample data from equipment providers, laboratories from previous terms, or materials freely available on the internet (e.g., frog calls). By using the provided data, the students could focus on the use of software to analyze data and perform statistical analysis. In some cases, we provided different groups with different data sets. In those cases, students analyzed the raw data, compiled data with other groups, learned and performed statistical analysis, and drew their conclusions. In other cases, we provided the same data set to different laboratory groups. For example, in a spirogram, students received the same data to analyze. They compared their numbers to see whether they had similar results or different results and whether they had analyzed the data the same way or differently. They then discussed the importance of interrater reliability. This exercise could be taken further by including discussions on the best way to present data and how to write an effective Results section.

When focusing on written and oral communication, the pros and cons of tables versus graphs, different types of graphs, or images of data (photos, screen shots) were discussed. This expanded to effective table or figure legend writing, the relevance of statistical measures, and how to use statistics when presenting data. Students then practiced compiling in-depth Results sections and Discussion sections. Although writing was a primary focus, presentation of data in posters versus oral presentations versus written documents was also discussed and practiced. As the scientific method is iterative, students were asked to develop a new hypothesis and research proposal based on the feedback received from the instructor and their peers. The addition of a peer review process as a component of the activity added a new dimension to the student learning process.

## THEME 3. USE FORMATIVE ASSESSMENT/REFLECTION TO MOTIVATE AND FACILITATE REMOTE LEARNING

As we used new strategies to connect with our students in a synchronous or asynchronous online format and revised the learning goals and objectives of the laboratory, it became obvious that our traditional assessment needed to evolve. Assessments drive learning, but often our existing assessments from in-person laboratories did not reflect learning objectives or fell behind the innovations we implemented in the laboratory. The limited ways to conduct traditional summative laboratory assessment in a remote setting have certainly exacerbated the situation. Additionally, in the online format instructors quickly recognized that the creation of assessments that are fair and equitable was difficult and time consuming and sometimes did not adequately reflect a student’s learning gains or their preparedness for a deeper understanding of course concepts. Moreover, the administration of closed-book quizzes/exams often required the use of tools (e.g., software, lockdown browsers) that could be distracting, cause unnecessary stress, and exacerbate inequalities. More troubling is that some learners have been reported to be at a disadvantage during remote assessment, because of unstable connectivity during exams, inability to view and revise previous questions after submission, and stress related to seeing the timer count down during timed quizzes, to name a few (23).

Given the current situation and the increasing mental health concerns for both students and instructors (24), we suggest that more weight should be given to formative assessment before, during, and even after online instruction, instead of a limited number of mid- or end-of-the semester summative exams. Frequent formative assessment has long been associated with an increase in learning gains (25). The group members found themselves favoring a course-based undergraduate research experience (CURE)-based assessment approach, where students are the drivers for inquiry, formative feedback is given throughout the program, and learning is assessed based on project outcomes and students’ abilities of communicating them rather than their ability to answer multiple-choice questions.

Formative assessments also increased the sense of belonging, trust, and self-motivation. From our experience, the primary role of frequent (formative) assessment was not about grades but rather about the creation of a dialogue between students, instructors, and their assistants, which guided course instructors on how to improve teaching and learning of course concepts in a remote setting. An added benefit of decreasing the weight of cumulative assessments (e.g., multiple-choice exams) in the final course grade is that it alleviated concerns about dishonesty and privacy (using lockdown or remote proctoring software) that are associated with these assessments.

Students can feel discouraged when studying in an isolated environment with less support available, as they are not always clear about what learning progress they have made. One notable practice that we introduced was to include time for reflection as an important component of building scientific skills. Below are ideas that we implemented:

- Regular low-stakes polling to assess learning but also interest and emotional standing (using student-response systems for polling or remote tools)
- Project-based inquiry (building up from low- to higher-order-level questions)
- Pointing out how the approaches used will also help in the short term in other classes and on standardized tests
- Testing on reading comprehension or analytical skills sections (social e-reading platform, such as Perusall)
- Mock interview responses that show how laboratory learning objectives are in line with professional career paths
- Ongoing open-ended reflections on what students can do or do better

As we progressed through the semester, it was evident that more emphasis needed to be placed on student motivation to achieve our learning outcomes and prevent disengagement. These assessment tools were used to incentivize students to buy into the nontraditional methods of instruction during remote learning. These newer methods will certainly become valuable assets when we return to teaching in-person physiology laboratories.

## CONCLUSIONS

In science education, laboratories remain a standard practice, in part because they serve as a recruiting device: the excitement of the laboratory often draws students into science (7). In addition, as noted above, laboratory enrollment is generally capped at 14–24 students, which often makes the laboratory the only place where students apply lecture concepts, may feel at ease to communicate and directly ask conceptual questions to a member of the teaching team, or find out more about research opportunities on campus. Because of this, we must pay close attention to retain the value of laboratories by continuing to motivate our students and allowing them to explore.

While the pandemic forced all disciplines to adapt their courses to this new platform, it created a unique opportunity for all instructors to rethink their existing curriculum. Clearly, the reflection and evaluation processes were and are different for each instructor. Nevertheless, for these authors, joining the NIST Physiology Lab Teaching Group during the pandemic (summer 2020) greatly facilitated the process. The pandemic made it difficult to develop students’ technical skills, but we saw this as a unique opportunity for laboratory instructors to focus on the development of executive skills. The most desirable gains our students achieved in executive skills were often centered around the scientific process and working as a team. Several instructors from our group have introduced/reintroduced formal laboratory reports as an integral part of laboratory exercises or, where more practical, have asked the students to complete only a section of a paper. Our favorite section has been the Results section, where students learn how to analyze the raw data, apply statistical analysis, find ways to demonstrate the trend, and summarize the results. We found that this approach can be an impactful learning experience.

Many physiology laboratories are cotaught by multiple instructors and/or teaching assistants and are facilitated by peer-learning assistants. When introducing any innovative approach in a team-teaching setting, the instructors need to reach agreement that the changes are beneficial, necessary, and achievable, as new pedagogical emphases and new practices might not be readily understood or appreciated by the entire instructional team. Ideally, when all teaching team members buy into the practices and ideas, the practices across different sections will become consistent and the intended learning outcomes will be most likely achieved. Therefore, it is important to provide adequate training and reflection opportunities for all teaching team members. When we designed our instructor/assistant training, we found it beneficial to create opportunities for all instructors to voice their concerns, brainstorm solutions collectively, and provide reflection moments as the semester progresses.

Although the desire to move to open inquiry was present, many challenges remained. Indeed, the design of assignments that effectively assessed and guided inquiry needed careful scaffolding, which increased grading time. The creation of instructions and resources was needed for students to develop the necessary skills for more open inquiry. Training and coordination of the instructional staff were required to guide more open inquiry, as they interacted with students informally and through assessments. Teams of students needed to be built so they could work together independently and inclusively. Other challenges with multiple scaffolded assignments included a much more varied set of student products to be graded and predicted time management challenges for students, as they navigated designing, performing, and analyzing their own experiments. In cases where the goals were more skill oriented, as in courses designed for students entering health careers, the goal was not to move toward more open inquiry but to create alternative ways of best meeting the objectives of technical skill development. We found COVID-19-proof alternatives for some, but not all, of our goals.

What about the content that was omitted? Not all basic science skills can be included in an online setting. Students who were trained under current circumstances will likely require some additional training to be adequately prepared for their careers. It is important that each program thoroughly reviews their curriculum maps, determines what skills are missing from current offerings and where they could be made up, and communicates those findings with upper-level instructors and program directors. Student heterogeneity will increase regardless of our best efforts, as not all students will take these courses or may enroll at other institutions. We are convinced, however, that by temporarily stepping away from our teaching routines we have successfully reintroduced much-needed fundamental training in building scientific proficiency and identity.

Despite all the setbacks imposed by the pandemic, it has further pushed us to be more creative and to embrace the focus of asking testable questions and devising studies to address them. Elements of our current online teaching, such as strategies for engaging all students, are likely to remain as important when we return face-to-face instruction. Large-scale data set sharing and community-based research, such as CitizenScience.gov, have opened doors for developing new lesson plans that lead to direct contributions to the science community, foster engagement, and result in motivation gain and agency development. New curriculum development and sharing have become easier, as collaborative tools and online meetings have made collaborations across different institutions and disciplines commonplace. The generous thinking that drives these collaborative efforts has also provided much comfort and energy to instructors. Quantitative Undergraduate Biology Education and Synthesis (QUBES) has published COVID-19-related lessons that focus on quantitative analysis. Organizations such as the Professional and Organizational Development (POD) network and the American Society for Microbiology (ASM) have curated and shared extensive resources for developing online laboratories. The National Institute on Scientific Teaching (NIST) has hosted many happy hours to showcase best teaching practices in online settings and facilitated working groups like ours. As observed in other fields, the strengthening of collaborations among instructors working in STEM is crucial as improved coordination across institutions can give clear guidance based on lessons learned, reduce inequities, and ultimately benefit our students (26). Therefore, although the return to in-person physiology laboratories is highly desirable, a full reversal to the prepandemic teaching format that often favored top-down approaches with fixed step-by-step instructions is unlikely. This is because many physiology instructors (including us) were forced to implement new instructional approaches amenable for the online delivery of most of the laboratory learning objectives. In turn, some of these new approaches proved to be effective strategies to implement collaborative/inclusive work regardless of the course setting, making the current disruption to structured curricula a transformative learning experience—not only for students but also their instructors—that has inspired the way we design our laboratories today and for years to come.

## GRANTS

C.B.K.-S. is supported by an Improvement of Teaching grant from the Widener University Provost’s Office. K.L.M. is supported by National Science Foundation (NSF) Grant 1524878. T.v.d.M. is supported by start-up funds from the University of California, Davis. X.C. is supported by NSF ECR-HER 2000613 and RCN-UBE 1827108.

## DISCLOSURES

No conflicts of interest, financial or otherwise, are declared by the authors.

## AUTHOR CONTRIBUTIONS

X.C., C.B.K.-S., T.v.d.M., K.L.M., A.H.S., and M.A.F.-D. prepared figures; drafted manuscript; edited and revised manuscript; and approved final version of manuscript.
